# Overview and Strategy Analysis of Technology-Based Nonpharmacological Interventions for In-Hospital Delirium Prevention and Reduction: Systematic Scoping Review

**DOI:** 10.2196/26079

**Published:** 2021-08-26

**Authors:** Chan Mi Kim, Esther M van der Heide, Thomas J L van Rompay, Gijsbertus J Verkerke, Geke D S Ludden

**Affiliations:** 1 Department of Design, Production, and Management Faculty of Engineering Technology University of Twente Enschede Netherlands; 2 Patient Care and Monitoring Department Philips Research Eindhoven Netherlands; 3 Department of Communication Science Faculty of Behavioral, Management, and Social Sciences University of Twente Enschede Netherlands; 4 Department of Biomechanical Engineering Faculty of Engineering Technology University of Twente Enschede Netherlands; 5 Department of Rehabilitation Medicine University of Groningen University Medical Center Groningen Groningen Netherlands

**Keywords:** intensive care unit, delirium, delirium prevention, delirium reduction, delirium treatment, technology, technology-based intervention, strategy, nonpharmacological, systematic scoping review

## Abstract

**Background:**

Delirium prevention is crucial, especially in critically ill patients. Nonpharmacological multicomponent interventions for preventing delirium are increasingly recommended and technology-based interventions have been developed to support them. Despite the increasing number and diversity in technology-based interventions, there has been no systematic effort to create an overview of these interventions for in-hospital delirium prevention and reduction.

**Objective:**

This systematic scoping review was carried out to answer the following questions: (1) what are the technologies currently used in nonpharmacological technology-based interventions for preventing and reducing delirium? and (2) what are the strategies underlying these currently used technologies?

**Methods:**

A systematic search was conducted in Scopus and Embase between 2015 and 2020. A selection was made in line with the Preferred Reporting Items for Systematic reviews and Meta-Analyses extension for Scoping Reviews (PRISMA-ScR). Studies were eligible if they contained any type of technology-based interventions and assessed delirium-/risk factor–related outcome measures in a hospital setting. Data extraction and quality assessment were performed using a predesigned data form.

**Results:**

A total of 31 studies were included and analyzed focusing on the types of technology and the strategies used in the interventions. Our review revealed 8 different technology types and 14 strategies that were categorized into the following 7 pathways: (1) restore circadian rhythm, (2) activate the body, (3) activate the mind, (4) induce relaxation, (5) provide a sense of security, (6) provide a sense of control, and (7) provide a sense of being connected. For all technology types, significant positive effects were found on either or both direct and indirect delirium outcomes. Several similarities were found across effective interventions: using a multicomponent approach or including components comforting the psychological needs of patients (eg, familiarity, distraction, soothing elements).

**Conclusions:**

Technology-based interventions have a high potential when multidimensional needs of patients (eg, physical, cognitive, emotional) are incorporated. The 7 pathways pinpoint starting points for building more effective technology-based interventions. Opportunities were discussed for transforming the intensive care unit into a healing environment as a powerful tool to prevent delirium.

**Trial Registration:**

PROSPERO International Prospective Register of Systematic Reviews CRD42020175874; https://www.crd.york.ac.uk/prospero/display_record.php?RecordID=175874

## Introduction

### Background

Delirium is an acute brain dysfunction with a disturbance in attention, awareness, and cognition [1], which is common, especially in critically ill patients. It occurs in about 40% of patients in the intensive care unit (ICU) [2], and in case of ventilated patients, the proportion goes up to about 65%-80% [2,3]. Nonetheless, delirium can be often underestimated due to differences in the severity of illness in populations and underrecognition of delirium [4,5]. Delirium is frequently associated with a significant increase in the ICU length of stay [6], risk of long-term cognitive impairments [7], and 6-month mortality rates after leaving the ICU [8]. At the organizational level, delirium is associated with an increase in the cost of ICU care [9], ICU readmission [10], and mental stress of ICU nurses who take care of patients with delirium [11]. At the societal level, delirium costs up to $152 billion per year in health care in the United States [12]. Prevention of delirium may be the most effective way to avoid these negative outcomes. In case of in-hospital patients, at least 30%-40% of delirium cases are preventable by reducing the risk factors of delirium [13,14]. There are 2 types of interventions used for preventing delirium: pharmacological and nonpharmacological interventions (eg, reorientation).

Nonpharmacological multicomponent interventions are recommended over pharmacological interventions as a safe and promising way of preventing delirium [15]. Nonpharmacological multicomponent interventions aim at reducing delirium risk factors that are modifiable by, for instance, promoting better sleep, early mobilization, and cognitive training/stimulation [15]. In order to support the implementation of nonpharmacological interventions, several methods [16,17] have been introduced. As an example, the ABCDEF bundle provides practical ways to provide optimal care of ICU patients [16]. Despite the benefits of using these methods [18], there are still barriers, as the way in which nonpharmacological interventions are applied is often highly dependent on the medical staff. Technology-based interventions might help to overcome these barriers. In this review, “technology” is defined as equipment that is used or developed based on scientific knowledge for practical purposes. Ironically, while the ICU room has been one of the most technology-intensive places in hospitals, when it comes to delirium prevention, the use of technology-based interventions in the ICU room often remains limited to, for example, the use of earplugs [19].

With recent developments in technology, there is large potential for the diversity of technology-based interventions. Examples include a modified ICU room design providing, among others, a personalized light therapy system and noise reduction to support cognitive stimulation and a normal sleep-wake cycle of patients [20]. Another example is an interactive app using a conversational agent, which provides patients social interactions and medical advice [21]. The app enables personalized care for patients without frequent physical visits of bedside nurses. These examples showcase some of the possibilities that recent developments in technology can bring. However, understanding how to optimize technology-based interventions is limited, and therefore, it is worthwhile to further and more systematically study the topic.

### Prior Work

Recently, several (systematic) reviews have been performed to provide overviews and to highlight the potential of existing nonpharmacological interventions for preventing delirium [18,22-28]. However, none of the existing reviews focused exclusively on technology-based interventions for preventing delirium. Moreover, none of the previous reviews [25-27] provided information necessary for improving the design and development of new technology-based interventions. For instance, a vision outlining what is needed to build a better care environment for patients in the context of intensive care and clear guidance for technology-supported improvements are lacking.

### Goal of This Study

To close this gap, this paper will provide a comprehensive review of technology-based interventions used for preventing and reducing in-hospital delirium. To our knowledge, this is the first systematic effort to provide such an overview. Taking a bottom-up approach, we will first investigate what types of technologies are used in current nonpharmacological interventions and how these interventions contribute to delirium prevention and reduction in hospitalized patients. Second, we will identify underlying strategies of technologies applied in the technology-based interventions. Finally, following our analysis, we will discuss the limitations of the current technology use and the opportunities for further research and development of technology-based interventions aimed at delirium prevention in the ICU and for other hospitalized patients. This systematic scoping review was conducted to answer the following questions: (1) what are the technologies currently used in nonpharmacological technology-based interventions for preventing and reducing in-hospital delirium? and (2) what are the strategies underlying these currently used technologies? This review will provide insights into technologies that can be used for in-hospital delirium prevention and reduction and suggest directions for future design and development of innovative technology-based interventions. We propose that incorporating these insights will optimize the use of technologies and enhance the effectiveness of technology-based interventions.

## Methods

### Search Strategy

This systematic scoping review was conducted complying with the Preferred Reporting Items for Systematic reviews and Meta-Analyses extension for Scoping Reviews (PRISMA-ScR) guidelines [29]. The study protocol was registered on the international prospective register of systematic reviews (PROSPERO, CRD42020175874). The search was conducted from the Scopus and Embase databases for studies that are written in English and were published between January 1, 2015 and January 6, 2020 (Scopus)/January 13, 2020 (Embase). This time period was set to focus on the state-of-the-art technologies used following our scope for this review. The initial search was conducted with the terms delirium (deliri*) and technology (technolog*) but it did not result in a sufficient number of relevant studies. Samples of search terms were identified through previous studies and extended through the search of index terms, medical subject headings, and other technological terms used for other medical purposes. The following terms were used for the final search: (1) deliri*, (2) technolog*, (3) intelligen* OR automat* OR digital* OR computer OR computing OR robot*, (4) mobile OR app, (5) visual OR virtual OR VR OR video, (6) light* OR ambien* OR aroma* OR architect*, (7) sound* OR music* OR voice OR alarm, (8) cognitive training OR tracking OR game*, and (9) 1 AND 2 OR 3 OR 4 OR 5 OR 6 OR 7 OR 8 (see Multimedia Appendix 1 for the full search strategy)*.* Additional studies were sought through communication with experts. Only studies that were peer reviewed and conducted with human subjects were included. The title-abstract screening and the full-text review were performed by 2 reviewers (CMK and EMvdH) independently. Disagreements were discussed and reevaluated based on the main goal of the study. The reasons for excluding were recorded.

### Eligibility Criteria and Study Selection

The studies were included based on the eligibility criteria (See Table 1).

**Table 1:** Eligibility criteria.

Criterion	Inclusion criteria	Exclusion criteria
Study type	All types of original research published in peer-reviewed journals and conferences	Letters, comments, editorials, conference abstracts, or any type of review
Period	January 1, 2015 until January 6 (Scopus) and 13 (Embase) 2020	Before January 1, 2015 and after January 6 (Scopus), 2020 and January 13 (Embase), 2020
Language	English	All other languages
Population	In-hospital patients in all age groups	Patients who are not in-hospital patients, healthy participants
Intervention	Nonpharmacological interventions using any type of technology either as a single intervention or as a part of multicomponent intervention aiming at preventing or reducing delirium	Pharmacological interventions, interventions only focusing on detecting/screening delirium
Comparator	Any comparator, including no comparator	N/A^a^
Outcome	Delirium-related data (eg, occurrence, duration of delirium) or risk factor–related data that indirectly influence delirium (eg, anxiety)	Neither delirium-related data nor risk factor–related data

^a^N/A: not applicable.

The main goal of this review was to find papers dealing with the prevention and reduction of delirium in the ICU, but in order to not overlook the potential of a greater range of technology-based interventions, the search scope was not limited to the ICU department but included also other hospital departments (eg, pediatric ICU, geriatric ward). We also included delirium from all age groups: from acute pediatric delirium to geriatric delirium because they share a similar range of delirium symptoms [30]. Moreover, the risk factors and recommended interventions across delirium in these groups are more or less the same [31]. Although delirium consists of 3 subtypes, each of which have their own symptoms and courses [32], these subtypes were not applied in the eligibility criteria and the study selection, as most studies did not specify them. We included studies focusing on incident delirium as well as prevalent delirium in order to address the full scope of delirium interventions. As we intended to explore all existing technology-based interventions supported by scientific evidence, we did not place any inclusion restrictions on the study design.

### Data Extraction and Quality Assessment

Data extracted from the studies included the primary author, year of publication, country of origin, publisher, summary of intervention content, applied technology, intervention goal, study design, type and number of participants, outcome measure, intervention outcomes, key findings, and limitations of the study. The primary data extraction was performed by CMK using the predesigned data extraction form, and the extracted data were reviewed and confirmed by EMvdH. Disagreements were resolved by discussions between CMK and EMvdH, which included revisits of the relevant data by both authors. To distinguish differences in the strength of evidence in the studies, a quality assessment was performed by CMK using a predesigned assessment form (Multimedia Appendix 2) and reviewed and confirmed by EMvdH. Disagreements were resolved by follow-up discussions. However, none of the studies were excluded based on the quality assessment in our analysis as our goal in this review was to create an overview of the existing technology-based interventions.

### Data Analysis

First, all technologies used in the technology-based interventions were looked into and clustered per technology type; a total of 8 categories was identified. Clustering was performed by CMK and reviewed and confirmed by EMvdH. Next, to summarize the strategies used in the technology-based interventions, goals and content summaries of each intervention were looked into, and 14 strategies were identified. These strategies were clustered and labelled based on the overarching theme: this led to the identification of 7 pathways to delirium prevention. An extraction of 14 elements from each intervention was carried out by CMK and clustering was performed independently by CMK and 2 other experienced researchers. After each session, the results and disagreements were discussed by CMK with the other experienced researchers, and then the synthesis was finalized by CMK.

## Results

### Study Selection

**Figure 1:**
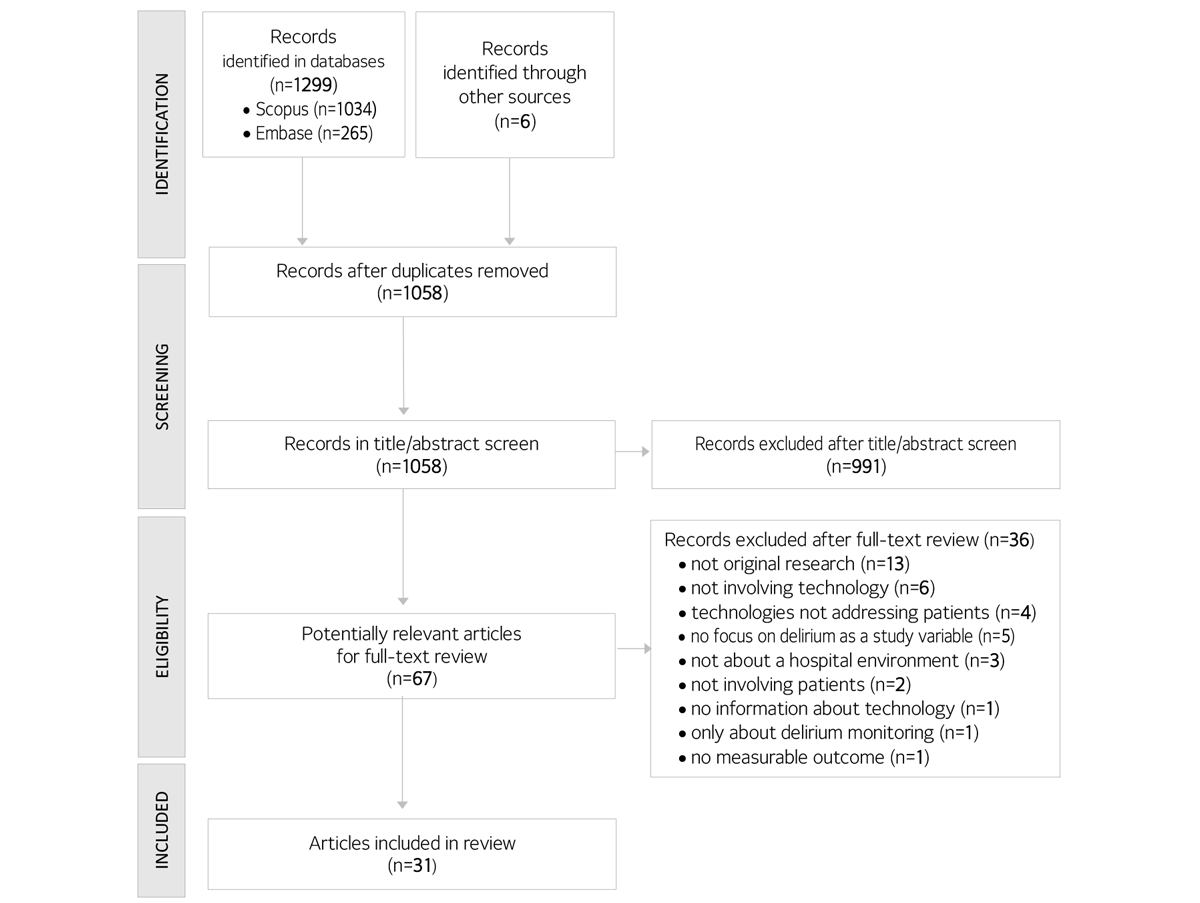
Flow diagram of study selection.

The process of literature screening and selection is presented in the flow diagram (Figure 1). After removing the duplicates, a total of 1058 studies were screened. Among these, 67 studies were examined based on the full text and 31 studies met inclusion for the review.

### Study Characteristics

The characteristics of the 31 included studies are listed in Table 2. Most of the studies were conducted in the ICU (15/31, 48%) followed by hospital wards (13/31, 42%) and both ICU and hospital wards (3/31, 10%). The most common study population was adult patients (23/31, 74%). Seven studies were conducted with pediatric patients [33-39] and 1 study was conducted with both adult and pediatric patients [40]. Most studies were prospective studies (28/31, 90%), including randomized trials [33-49], pretest-posttest/experimental [19,21,24,50-52], organizational case study [53], observational cohort [54], and pilot studies [55-57]. There were 3 retrospective studies [58-60] (See Table 2 for further specifications).

**Table 2:** Characteristics of the included studies classified into 8 different technology types.

Technology type, study			Main goal of intervention		Study design		Patient type^a^		Total number of patients (per group)
**Audio: music/voice message**									
	Damshens et al [40]	To improve mental state^b^		Randomized clinical trial		ICU^c^ (pediatric and adult)		80 (I^d^: 40, C^e^: 40)	
	Lee et al [39]	To reduce stress and anxiety		Randomized controlled trial		ICU		85 (I: 41, C: 44)	
	Johnson et al [48]	To alter physiologic response		Randomized controlled trial		ICU/trauma orthopedic unit		40 (I: 20, C: 20)	
	Sharda et al [53]	To mitigate postoperative pain and anxiety^b^		Organizational case study		Perioperative optimization of senior health		202 (I: 45, C: 157)	
	Cheong et al [55]	To enhance engagement and mood and to improve agitated behavior^b^		Pilot study (for randomized controlled trial)		Acute care unit (patients with delirium, dementia, or both)		25 (I: 25, C: 25)	
	Byun et al [33]	To activate positive psychological and behavioral responses and to reduce anxiety^b^		Double-blind randomized controlled trial		PACU^f^ (pediatric)		66 (I1: 33, I2: 33)	
	Munro et al [49]	To support reorientation and to comfort patients^b^		Three-group prospective randomized controlled trial		ICU^g^		30 (I1:10, I2:10, C: 10)	
**Light: Dynamic light/natural light**									
	Estrup et al [58]	To improve circadian rhythm^b^		Retrospective cohort study		ICU		183 (I: 46, C: 137)	
	Pustjens et al [60]	To improve circadian rhythm^b^		Retrospective observational cohort study		Coronary care unit		748 (I: 369, C: 379)	
	Simons et al [41]	To improve circadian rhythm and sleep^b^		Randomized controlled single-center trial		ICU		734 (I: 361, C: 373)	
	Potharajaroen et al [42]	To improve sleep-wake cycle^b^		Single-blind randomized controlled study		ICU		62 (I: 31, C: 31)	
	Smonig et al [54]	To reduce circadian rhythm disruption^b^		Prospective single-center observational study		ICU		179 (I: 102, C: 77)	
**Video/video game: information/distraction**									
	Lee et al [50]	To reduce preoperative anxiety^b^		Quasi-experimental		ICU		50 (I: 25, C: 25)	
	Kim et al [34]	To reduce preoperative anxiety		Prospective randomized controlled trial		PACU (pediatric)		104 (I1: 34, I2: 33, I3: 37)	
	Rodriguez et al [35]	To reduce preoperative anxiety		Prospective randomized trial		PACU (pediatric)		52 (I1: 25, I2: 27)	
	Waszynski et al [43]	To reduce agitation in patients experiencing hyperactive or mixed delirium		Randomized controlled trial		Hospitalized/ICU^h^		111 (I1: 34, I2: 40, C: 37)	
	Dwairej et al [36]	To reduce preoperative anxiety		Randomized clinical trial		Day case surgery unit (pediatric)		128 (I: 64, C: 64)	
**Virtual reality: information/distraction**									
	Eijlers et al [37]	To reduce pain and anxiety^b^		Randomized controlled single-blind trial		Day case surgery unit (pediatric)		191 (I: 94, C: 97)	
	Ryu et al [38]	To reduce preoperative anxiety		Randomized controlled trial		PACU (pediatric)		86 (I: 41, C: 39)	
	Suvajdzic et al [57]	To reduce clinical anxiety and depression and to support sleep and relaxation^a^		Pilot test		ICU		10	
**Sleep aids**									
	Demoule et al [44]	To improve sleep quality		Randomized controlled trial		ICU		45 (I: 23, C: 22)	
	Van de Pol et al [19]	To improve sleep quality		Interrupted time series design (preintervention and postintervention)		ICU		421 (I: 210, C: 211)	
**Communication aids**									
	Garry et al [56]	To improve patient’s psychosocial status^b^		Pilot prospective trial		ICU/coronary care unit		12	
	Bott et al [21]	To provide companionship, social support, and health information to decrease loneliness and depression^b^		Case control quasi-experimental pre-post (for randomized controlled trial)		Medical and surgical unit		95 (I: 41, C: 54)	
**Others**									
	Lin et al [46]	To make the children adapt to the experience of visual disturbance		Prospective blinded randomized trial		PACU (pediatric)		179 (I: 89, C: 90)	
	Giraud et al [45]	To support mental status, earlier physical mobilization, and multisensory feedback/integration		Pilot time-cluster randomized controlled study		ICU		223 (I: 115, C: 108)	
**Multiple components**									
	Arbabi et al [51]	To support reorientation, cognitive/physical activation, human interaction^b^		Quasi-experimental		ICU		148 (I: 78, C: 69)	
	Tovar et al [52]	To reduce environmental precipitating factors, which impair sleep and to support maintenance of circadian cycle.		Prospective pre-experimental		ICU		49	
	Rivosecchi et al [24]	To provide cognitive stimuli and to support reorientation^b^		Prospective, observational quality improvement project pre-post		Medical ICU		483 (I: 253, C: 230)	
	Mitchell et al [47]	To support orientation and to provide cognitive stimulation		Single center randomized controlled trial		ICU		61 (I: 29, C: 32)	
	Zachary et al [59]	To prevent functional/cognitive, sleep and visual/hearing impairment, and dehydration		Retrospective		Medical, surgical/telemetry units^i^		4850 (I: 2146, C: 2704)	

^a^All patients are adults unless mentioned as pediatric patients.

^b^With our minimal interpretation.

^c^ICU: intensive care unit.

^d^I: intervention group.

^e^C: control group.

^f^PACU: post anesthesia care unit.

^g^About 10% was delirious at admission.

^h^Only delirious patients.

^i^Excluding intensive care unit patients.

### Quality Scores of the Included Studies

The assessed quality scores ranged from 1 [24,56] to 5 [32,36-39,46,48] (Multimedia Appendix 2). The average score was 3.258 (SD 1.264). A score lower than 3 was found in 10 studies [21,24,51-53,55-58,60]. Seven studies reached the maximum score of 5 [33,36-39,46,48]. The included studies were analyzed in 2 ways: first, by focusing on extracting the different types of technologies in the interventions, and second, by focusing on identifying strategies underlying these interventions.

### Types of Technologies Currently Used for Preventing Delirium

A total of 31 technology-based interventions were identified (Table 2 and Table 3). Eight categories were distinguished based on the type of technology used. These categories are audio (7 studies), light (5 studies), video/video game (5 studies), virtual reality (VR) (3 studies), sleep aids (2 studies), communication support (2 studies), and others (2 studies). Technologies used as part of a multicomponent approach were grouped into a category called multiple components (5 studies). The identified technologies varied from simple (eg, earplugs, window blinds) to more advanced (eg, dynamic light, VR) ones. These technologies were used to complement conventional delirium treatments rather than replace them by reducing the negative (psychosocial) consequences of environmental factors and their effects on patient experiences.

**Table 3:** The content and effects of technology-based nonpharmacological interventions.

Study		Summary of the intervention	Incidence of delirium, *P* value	Duration of delirium (days)	Effectiveness		Key findings
					Direct^a^	Indirect^b^	
**Audio: music/voice message**							
	Damshens et al [40]	*Music therapy*: Listening to light instrumental music selected by a music expert for 45 minutes twice (once in the morning and once at night) for a day.	I^c^: 15 (37.5%) C^d^: 16 (40%) (*P*=.82)	N/A^e^	No^f^	Yes^g^	No significant difference between the control and test group in terms of incidence of delirium. Significantly lower use of 2 pain relievers (acetaminophen and diclofenac) in the test group.
	Lee et al [39]	*Music intervention*: A single 30-minute session of listening to slow-beat music (60-80 bpm) selected by patients from predefined playlists through headphones in the presence of a nurse at the bedside. The study was conducted to explore the anxiety-reducing effect of the intervention.	N/A	N/A	N/A	Yes	Music intervention significantly reduced anxiety and stress-related measures (serum cortisol level, heart rate, visual analog scale for anxiety, etc) of mechanically ventilated intensive care unit patients.
	Johnson et al [48]	*Music intervention*: Listening to simple repetitive, self-selected music with slow tempo (60-80 bpm), low pitch, and repetitive rhythms for 60 minutes using an iPod and headsets, twice a day, over 3 days following admission.	I: 0 C: 0	N/A	N/A	Yes	No delirium incidence in both the control and test group. Music intervention significantly improved the pathophysiological mechanisms that contribute to delirium, neurotransmitter imbalance, inflammation, and acute physiological stressors in the test group.
	Sharda et al [53]	*Confusion avoidance led by music program*: On postoperative day 1, patients got a music player and headphones with a personalized playlist based on a music assessment. Listening for at least 20 minutes twice a day was recommended, but patients had autonomy over the ultimate dose and frequency.	I: 17.8% C: 28.7% (*P*=.14)	N/A	No	No	This program may impact incident delirium and optimize postoperative pain and anxiety.
	Cheong et al [55]	*Creative music therapy*: Spontaneous music making with musical instruments, playing familiar songs of patient's choice and music listening for 30 minutes once a day over 2 days. Based on individual’s profile and response to music, a certified music therapist modified techniques to meet patients’ need.	N/A	N/A	N/A	Yes	This therapy can improve mood/emotion and engagement of patients with dementia and delirium.
	Byun et al [33]	*Mother's recorded voice*: Listening to either the recorded a voice of mother (I1) or a stranger (I2) through noise-cancelling headphones at the end of a surgery. The prerecorded message with standardized text was repeated with 10-second intervals and was continued until entering the postanesthesia care unit.	I1: 8 (24.2%) I2: 20 (60.6%) (*P*=.006)	N/A	Yes	N/A	Letting children listen to the sound of their mother in the recovery room reduced the incidence of emergence delirium.
	Munro et al [49]	*Automated reorientation*: During the first 3 days at the intensive care unit, patients either received automated reorientation messages with a familiar (I1) or unfamiliar voice (I2) or no reorientation message at all (C). The message containing patient’s name and information about the intensive care unit environment (<2 minutes long) was played during the day (9 AM to 4 PM).	Delirium-free day I1: 1.9 days (0.99) I2: 1.6 days (1.07) C: 1.6 days (1.13) (*P*=.04)	I1:0.3 (0.48) I2:0.6 (0.84) C: 0.9 (1.28) (not significant)	Yes	N/A	Reorientation through automated messages increased the number of delirium-free days. A familiar voice was more effective than an unfamiliar voice in reducing delirium.
**Light: dynamic light/natural light**							
	Estrup et al [58]	*Circadian light*: Exposure to the circadian light in which the amount of blue light (460-480 nm) changes over time like natural light. The light intensity varied from 50 lux to 4000 lux during daytime (between 6:00 and 20:30) and there was no blue light between 23:00 and 6:00. The exposure time of patients was varied from at least 24 hours to their total stay.	I: 30 C: 28	I: 2.5 (1-7) C: 1.5 (1-3) (*P*=.41)	No	N/A	Circadian light did not have an effect on the development of delirium.
	Pustjens et al [60]	*Dynamic light*: Exposure to an artificial daylight system with light intensity peak values of 750 lux at the eye level and a color temperature ranging from 2700 K to 6550 K. Patients were exposed as long as possible during daytime. The overall period varied from 20 hours to 42.7 hours depending on the patient.	I: 20 (5.4%) C: 19 (5.0%) (*P*=.80)	N/A	No	N/A	Exposure to dynamic light did not reduce the incidence of delirium nor total hospital length of stay.
	Simons et al [41]	*Dynamic light*: Exposure to the circadian light system with light intensity and color temperature peaks of 1700 lux and 4300 K via conventional fluorescent tubes between 9:00 and 16:00 except for 11:30 and 13:30 (intervention group) and to the standard lighting settings of 300 lux and 3000 K (control group) during the intensive care unit stay of patients (3-9 days).	I: 137 (38%) C: 123 (33%) (*P*=.16)	I: 2 (2-5) C: 2 (1-5) (*P*=.87)	No	N/A	Dynamic light as a single intervention did not reduce the cumulative incidence and duration of delirium in the test group.
	Potharajaroen et al [42]	*Bright light*: Treatment with bright light therapy consisting of 5000 lux at 1.4-m distance from patient’s face between 9:00 and 11:00 for 3 days. Other treatment data (nasal cannula oxygen, drugs etc) were analyzed.	I: 2 (6.45%) C: 11 (35.48%) (*P*=.005)	N/A	Yes	N/A	Bright light therapy reduced the incidence of delirium in the test group.
	Smonig et al [54]	*Natural light exposure:* Exposure to natural light via windows from admission to the intensive care unit until discharge (3-7 days).	I: 65 (64%) C: 55 (71%) (*P*=.28)	I:3 (1-6) C:3 (1-7) (*P*=.43)	No	Yes	Admission to a single room with natural light via windows did not reduce the incidence of delirium but a risk of agitation episodes and hallucinations
**Video/video game: information/distraction**							
	Lee et al [50]	*Preoperative video information*: Informative videos that explain preoperative procedures, operating room environment, and intensive care unit environment on the day prior to surgery.	I: 3 (12%) C: 5 (20%) (*P*=.26)	N/A	No	Yes	The intervention was not effective in reducing delirium incidence but decreased the anxiety levels.
	Kim et al [34]	*Video distraction and parental presence*: In the preoperative holding room before surgery, provision of a 4-minute animated cartoon video (I1), parental presence (I2), or a video plus parental presence (I3). The primary study goal was to compare the effect of video distraction, parental presence, or combination of both on the preoperative anxiety reduction.	I1: 13 (38.2%) I2: 13 (39.4%) I3^e^: 20 (43.5%) (*P*=.32)	N/A	No	N/A	All groups showed similar effect on the preoperative anxiety and none of them significantly reduced emergence delirium.
	Rodriguez et al [35]	*Video distraction using varying screen size*: Watching a movie in the preoperative area and through the induction of anesthesia. Patients chose from one of the 5 preselected age-appropriate movies using either a large bedside screen (I1) or a small tablet (I2). One parent accompanied a patient. The average time spent was 3.8 minutes (I1) and 4.5 minutes (I2). The primary study goal was to compare the effect of video distractions in different screen sizes on anxiety reduction.	I1: 29.16% I2: 30.8% (not significant)	N/A	No	Yes	The video distractions decreased the preoperative anxiety, regardless of the size, with parental presence at induction of anesthesia. No effect was found for the emergence delirium rate.
	Waszynski et al [43]	*Simulated family presence and nature scene*: Two types of video intervention for when agitation is present and the family is not: watching a 1-minute family video message plus usual care (I1) or watching a 1-minute nature video plus usual care (I2). The study goal was to examine the effect of family video message on agitation level.	N/A	N/A	N/A	Yes	Both family video message and nature video can decrease agitation in delirious patients.
	Dwairej et al [36]	*Video game distraction and anesthesia mask practice*: Combination of video distraction using a handheld video game (1-2 minutes) before the transfer to operation room, anesthesia mask exposure, and shaping intervention. During anesthesia induction, parental presence is allowed but not standardized. In the operating room, nonmedical talks occurred to distract the child. The study goal was to evaluate the effectiveness of the intervention on the preoperative anxiety.	I: mean 11.06, SD 3.97 C: mean 10.25, SD 4.81 (*P*=.30)	N/A	No	Yes	The intervention significantly reduced anxiety. Yet, the results did not reveal statistically significant difference in emergence delirium scores.
**Virtual reality: information/distraction**							
	Eijlers et al [37]	*Virtual reality exposure*: Provision of a 15-minute highly immersive virtual reality experience of the operating theatre to get familiarized with the environment and general anesthesia procedures. The virtual environment was computer-generated, interactive, and child-friendly.	I: 7.0 (5.0-9.0)^f^ C: 6.0 (5.0-9.0) (*P*=.27)	N/A	No	No	This did not have a beneficial effect on anxiety, pain, emergence delirium, or parental anxiety.
	Ryu et al [38]	*Preoperative immersive virtual reality tour of operating theater*: Provision of a 4-minute virtual reality video for pediatric patients showing the operating theater and explaining the perioperative process by using a popular animal character as a patient. The intervention was provided 1 hour prior to entering the operating room. The study goal was to examine the effect of the intervention on reducing the preoperative anxiety.	I: 16 (39%) C: 14 (36%) (*P*=.77)	N/A	No	Yes	The intervention did not reduce the incidence and severity of emergence delirium, although it was effective in alleviating preoperative anxiety in children.
	Suvajdzic et al [57]	*Patient-centered virtual reality system*: Intervention consisting of 2 sessions (session 2 was held at least 24 hours after session 1): (1) a video instructing patients to enjoy the movie by moving their heads to look around, followed by a 5-10-minute guided meditation in virtual nature scenes for breath control (Relax VR), (2) playing either Relax VR or fishing game (Bait!).	I: 0 (0%)	N/A	N/A	No	The interventions did not result in clinically significant changes in pain, sleep, or vital signs. It seems likely that greater exposure to virtual reality is more likely to produce a meaningful effect on patient physiology and sleep quality.
**Sleep aids**							
	Demoule et al [44]	*Earplugs and eye masks*: Use of earplugs and eye masks every night between 10 PM and 8 AM from inclusion until ICU discharge (average 7 days). The study goal was to evaluate the impact of the intervention on sleep architecture in intensive care unit patients.	I: 2 (7%) C: 2 (6%) (*P*>.99)	N/A	N/A	Yes	Interventions resulted in reduced long awakenings and increased deep sleep duration. Possibly the effect was at least partially counteracted by the discomfort of wearing the devices.
	Van de Pol et al [19]	*Nocturnal sound-reduction protocol*: A protocol focusing on reducing noise in the night, for example, speaking and laughing quietly in the lobby, minimizing alarm volume, closing the door when the patient is not delirious, and providing earplugs at night. One month of implementation phase.	Slope of delirium incidence I: –2.79% (*P*=.02) C: 0.91% (*P*=.37) Difference: –3.70% per time period (*P*=.02)	N/A	Yes	Yes	The protocol reduced the incidence of delirium. It significantly reduced delirium risk factors such as perceived nighttime noise and the use of sleep medication. Reported sleep quality was not improved.
**Communication aids**							
	Garry et al [56]	*Eye-tracking devices*: Usage sessions (45 min/session, 5 sessions on consecutive weekdays) were given, during which patients were prompted to spell out notes, indicate their needs via picture sets, and play simple memory games. Patients were permitted to communicate with family, nursing staff, and physicians outside the training sessions.	Day 1: 4 (33%) Day 2: 1 (8%) Day 3/onward: 0	N/A	N/A	Yes	The use of an eye-tracking device positively affected patients’ happiness and ability to participate; however, it did not show a significant effect on patients’ confusion level or frustration.
	Bott et al [21]	*Bedside digital care coach avatar*: 24-hour psychosocial and health care support through an embodied conversational agent with an appearance of animated animal avatar. It checks patient status, assists communication, and offers psychological support during their stay at medical and surgical units (3-6 days). The average time spent with the embodied conversational agent was 61 minutes per day.	I: pre12 (41%)/post1 (3%) C: pre6 (13%)/post3 (6%) (*P*<.01/.25)	N/A	Yes	Yes	The use of the care coach avatar during hospitalization can reduce the frequency of delirium, loneliness, and falls among diverse hospitalized older adults.
**Others**							
	Lin et al [46]	*Eyepatch for visual preconditioning*: Preventive treatment for pediatric patients undergoing ophthalmic surgery consisted of covering the eye with an eyepatch for at least 3 hours one day before surgery.	I: 15 (16.9%) C: 40 (44.4%) (*P*<.001)	N/A	Yes	Yes	The intervention significantly reduced preoperative anxiety and emergence delirium. Preoperative anxiety was found to be an independent risk factor of emergence delirium.
	Giraud et al [45]	*Structured mirrors intervention*: Protocol-driven mirrors intervention consisting of different mirrors to provide visual feedback about the environment as a reorientation tool and to support self-awareness and explanation of medical/nursing procedures. The unit of randomization was a 2-week time period cluster.	I: 20 (17%) C: 17 (16%) (*P*=.71)	I: 1 (IQR 1-3 [range 1-25]) C: 2 (IQR 1-8 [range 1-13]) (*P*=.40)	No	Yes	Use of the mirror intervention did not reduce delirium but improved factual memory encoding.
**Multiple components**							
	Arbabi et al [51]	*Environmental changes and liaison education*: Environment with proper time cues, appropriate lighting for the time of the day during intensive unit care stay (average about 5 days). Further, allowing interactions with family members and medical staff, giving vision and hearing aids, preventing dehydration, and encouraging early mobilization. Training for medical staff on delirium management.	I: 30 (37.97%) C: 50 (72.46%) (*P*=.01)	I: 26.18 (SD 35.38) C: 35.84 (SD 39.31) (*P*=.001)	Yes	N/A	Multifactorial intervention (educational and environmental changes) was effective in reducing the delirium rate in the intensive care unit and the duration of delirium.
	Tovar et al [52]	*Environment with reduced environmental stressors*: Nursing care guide to reduce environmental stressors such as noise and continuous artificial light. Provision of active interactions with family members and medical staff, cognitive/sensory stimuli, and information about the environment. The posttest was performed after the guideline had been applied for 5 days.	I: 3 (6.12%)	N/A	Yes	N/A	Multicomponent intervention focusing on reducing precipitating environmental factors was effective in reducing delirium incidence and improving sleep.
	Rivosecchi et al [24]	*Nonpharmacological protocol*: Nursing education bundled into the protocol consisting of music, opening blinds, reorientation and cognitive stimulation, eye and ear protocol during intensive care unit stay (median 188.3 hours-control group, and 153.5 hours-intervention group).	I: 24 (9.4%) C: 36 (15.7%) (*P*=.04)	I:16 h (IQR 8-24) C:20 h (IQR 9.5-37) (*P*<.001)	Yes	N/A	The protocol reduced both duration and incidence of delirium.
	Mitchell et al [47]	*Multicomponent family-delivered intervention*: Family intervention consisting of orientation (memory clues: family pictures etc), therapeutic engagement (cognitive stimulation: discussing current family life events etc), and sensory (glasses, hearing aids in place/working). The intervention group was enrolled for a median of 5 days and the family members were asked to deliver the intervention at least once a day. The study goal was to assess the feasibility and acceptability of the intervention for designing a larger randomized controlled trial.	I: 17 (59%) C: 18 (56%) (*P*=.87)	I: 1.0 (2) C: 1.0 (2) (*P*=.60)	No	N/A	Multicomponent family-delivered intervention was not effective in reducing delirium incidence nor days of delirium.
	Zachary et al [59]	*Hospital elder life program interventions*: Geriatric intervention program consisting of reorientation, social stimulation, music therapy, games, mindfulness relaxation, mobilization, visual/hearing aids, sleep aids, and nutrition support. Volunteer interventions were 3 times a day, which took 20-30 minutes each time.	N/A	N/A	N/A	Yes	This program reduced 30-day readmissions and hospital length of stay in the 70-85 years age group.

^a^Direct effects: incidence and duration of delirium.

^b^Indirect effects: length of stay or delirium risk factors, including but not limited to pain, anxiety, stress, mood, agitation, and sleep deprivation.

^c^I: intervention group.

^d^C: control group.

^e^N/A: not applicable.

^f^No significant positive effect.

^g^Significant positive effect.

Of the 31 identified studies, 23 studies analyzed the effect of the interventions on direct delirium-related outcome measures (incidence and duration of delirium). The other 8 studies only assessed indirect outcome measures (eg, length of stay, precipitating risk factors of delirium such as anxiety). Of the 23 studies that evaluated direct delirium-related outcome measures, 9 studies showed a significant effect on decreasing either or both incidence and frequency of delirium. The interventions in these studies involved diverse technologies across the categories, that is, audio [33,49], light [42], sleep aids [19], communication support [21], others [46], and multiple components [24,51,52]. Of the 14 studies without significant effect on direct outcome measures, 8 studies, however, showed significant effects on delirium risk factors or symptoms, including pain [40], anxiety [35,36,38,50], agitation [54], hallucination [54], and factual memory encoding [45]. Six of the 8 studies using diverse technology-based interventions showed a significant effect on indirect outcome measures, including anxiety [39], agitation [43], sleep deprivation [44], or ICU readmission [59]. The technologies used in these studies were audio [39,48,55], video/video game [43], sleep aids [44], and multiple components [59]. It is notable that across all technology categories, significant positive effects were found on either or both direct and indirect delirium outcome measures. An explanation and further analysis of each category is given as follows.

#### Audio

The interventions using audio had either music or prerecorded voice messages. Music interventions involved, in general, slow tempo music with a duration of 20 minutes to 1 hour. The music was provided 2 or 3 times a day by using a television audio system or headset. These interventions aimed at addressing anxiety [39,53], mood [55], pain [40,53], and engagement [55] for adult patients. Across these interventions, we found differences in the level of personalization and autonomy: the level of personalization varied from the provision of music preselected by a music expert to the creation of a music playlist based on a patient’s own choice and the level of autonomy ranged from patients listening to music with dose and frequency predecided by a researcher to patients deciding on dose and frequency. Interestingly, delirium incidence was reduced in the study with personalized music allowing patients autonomy [53]. While the majority of studies used a passive format such as music listening, 1 study used a participatory format providing an interactive music therapy and showed significant improvement in mood and engagement [55]. Prerecorded voice messages contained mainly patient’s name and information about the care environment using either a familiar or unfamiliar voice. These interventions aimed to provide reorientation and a feeling of comfort. Two studies were carried out with different patient age groups: one with pediatric patients and the other with adult patients. The use of a prerecorded voice message reduced direct delirium outcome in both groups [33,49]. Likewise, one of the studies showed that a familiar voice was more effective than an unfamiliar one [49].

#### Light

The interventions involving light used either a specially made light therapy system for dynamic/bright light or natural light through windows. Dynamic light interventions provided light consisting of diverse intensities (ranging from 50 lux to 4000 lux) and color temperatures (ranging from 2700 K to 6550 K) in the environment of patients [41,58,60]. The bright light intervention used high intensity of light (5000 lux) for 2 hours a day [42] while the natural light intervention used natural light coming through windows [54]. All studies aimed at improving the patients’ circadian rhythm and were only tested on adult patients. Only the bright light intervention showed an effect on reducing the incidence of delirium [42]. Possibly, most of the current light interventions are not effective in reducing direct delirium outcomes. Another possible explanation for why few studies found an effect on reducing incidence of delirium might relate to the amount of light (intervention) that patients actually received. In most studies, for example, the actual light intensity at patients’ eye level was not specified or the definite exposure time to light of each patient was not guaranteed as sedative patients were included who had their eyes closed [54,58,60].

#### Video/Video game

The interventions using video/video games were used for providing either information about the medical procedure prior to a surgery or distractions with various contents, including age-appropriate programs (eg, cartoons), family messages, or nature scenes through a handheld tablet or a specially made bedside screen. These interventions aimed to address anxiety or agitation and were effective in decreasing anxiety or agitation in all studies with either pediatric or adult patients [34-36,43,50]. Regarding direct delirium outcome measures, none of the studies showed a significant effect [34-36,50].

#### VR Technology

The interventions using VR technology provided either information about medical procedures, distraction, or sensory stimulation for patients. VR interventions used a head-mounted device and the VR content varied from a guided tour to the operating theatre, to the virtual scenes of real-world locations. The interventions were mainly used to address anxiety or to support restorative effect for both pediatric and adult patients [37,38,57]. The effects of using VR to provide information (a preoperative VR tour to the operating theatre) differed in the studies. Of the two, 1 study showed a significant effect on reducing the anxiety of pediatric patients [38]. For none of these studies, the use of VR resulted in any effect on delirium-related outcome measures [37,38,57].

#### Sleep Aids

The interventions related to sleep aids were wearable devices such as earplugs and eye masks [19,44], and environmental modifications such as closing doors and window blinds were applied [19]. They aimed at reducing noise and light during nighttime to improve the sleep quality of adult patients. The application of both wearables and environmental modifications showed an effect on reducing the incidence of delirium [19]. Moreover, the use of a wearable showed an improvement in sleep quality [44]. It is notable that this study also highlights the potential negative side-effects of using wearables due to discomfort experienced by vulnerable patients.

#### Communication Supports

The technologies used for communication supports were a conversational agent [21] and an eye-tracking device [56]. They assisted adult patients to express their needs or to participate in psychosocial activities. Both types improved the psychological well-being of patients by increasing happiness and the ability to participate and by reducing loneliness. The conversational agent was used as a digital care coach providing communication means, human interactions, and companionship. This intervention reduced the frequency of delirium [21].

#### Others

In this category, we found rather simple forms of technologies used for diverse purposes: an eyepatch for experiencing what will happen after surgery [46] and a structured mirror to provide patients cognitive stimuli as a means to support mobilization and communication [45]. The eyepatch was used for pediatric patients and was effective in reducing emergence delirium [46]. The structured mirror intervention was for adult patients and improved their factual memory encoding [45].

#### Multiple Components

Some interventions involved more than one technology as part of a nonpharmacological bundle for adult patients. Environmental modifications for noise reduction, cognitive stimulation, and reorientation reduced delirium incidences in 3 studies [24,51,52]. Zachary et al [59] had a similar focus and showed an effect on reducing the 30-day readmission. In a small feasibility study, no significant effect was found on direct delirium outcome measures for the simple technology-based intervention involving families throughout different therapies such as orientation and cognitive stimulation [47].

### Seven Pathways to Delirium Prevention

Fourteen strategies to prevent delirium were identified from the technology-based interventions of the included studies (see Multimedia Appendix 3 for the 14 strategies used in the included studies and see Table 4 for full description of the 14 strategies).

**Table 4:** Descriptions of the 14 strategies.

Strategy	Explanation	Example
Cognitive stimulation and training	Stimulating patient’s brain activity to maintain and improve their cognitive capability and executive functions such as attention, memory, reasoning, and language.	Music, book
Companionship	Providing patients a sense of consistent social presence as a means to combat social isolation and loneliness.	Digital agent
Contextual cue (reorientation)	Providing patients contextual information such as time, date, and place to minimize confusion and anxiety coming from not knowing what’s going on and feeling lost	Automated voice message, clock
Daytime awakening	Supporting patients to stay physically and mentally activated during day so they can become tired enough to sleep at night	Dynamic light
Distraction	Redirecting patient’s focus away from distressing situations/conditions such as pain, discomfort, fear, and anxiety	Music, video, virtual reality
Early mobilization	Encouraging patients to move their bodies early enough to prevent muscle loss and other complications caused by lack of physical movement	Structured mirror
Easier communication	Providing a means for patients to better express their needs especially when they are mechanically ventilated	Eye-tracking device
Engagement	Encouraging patients to be interested in and to be involved with what is happening	Participatory music therapy
Familiarity	Providing something that patients feel familiar with to help them feel safe, at ease, and calm	Mother’s voice, personalized music list
Good night sleep	Providing an environment that facilitates sleeping by removing disturbing elements such as sound and light noise and by adding elements enhancing patient comfort and relaxation	Ear plugs
Human (social) interaction	Providing patients warm human (-like) interactions to stimulate them socially and to help them feel being involved and being cared for	Digital agent
Personalization	Providing an option that reflects patient’s preference	Personalized music list
Psychological preparation	Helping patients to feel prepared and confident by informing them what will happen in advance.	Virtual tour to the operation room
Soothing elements	Helping patients to calm down and to manage stress and anxiety by providing an activity or environment that is soothing	Music, nature video

**Figure 2:**
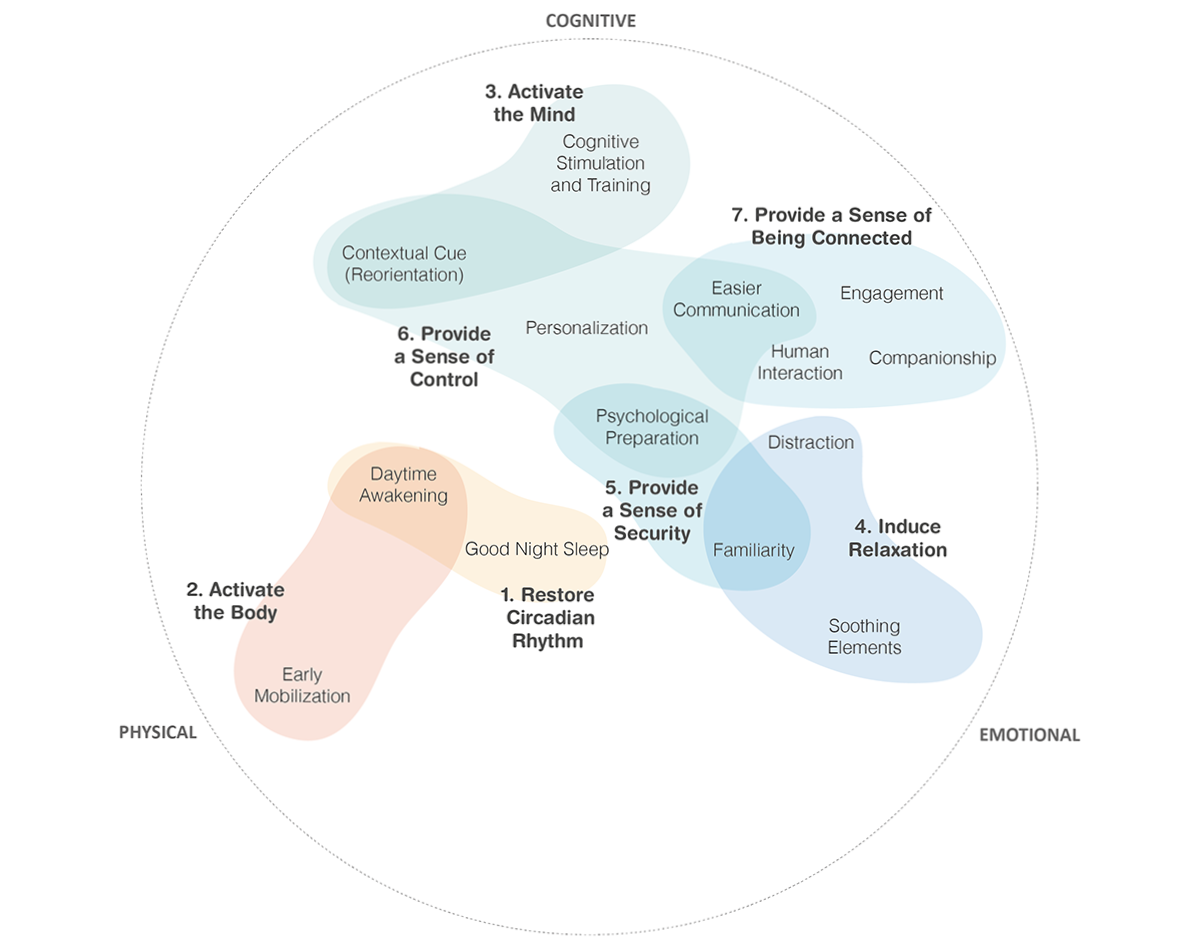
Overview of the 14 strategies grouped into 7 pathways of delirium prevention that are used in technology-based interventions.

Subsequently, the 14 strategies were clustered into 7 pathways by using a thematic analysis approach (Figure 2). As such, the 7 pathways might provide directions toward technology-based interventions for delirium prevention. The 7 pathways and a short description of each pathway are as follows:

1. Restore the circadian rhythm: helping patients to find a normal sleep-wake cycle to prevent sleep deprivation.
2. Activate the body: supporting patients to regain physical strength and endurance.
3. Activate the mind: supporting patients to prevent cognitive decline, restore cognitive function, and minimize confusion, which can cause negative emotions such as anxiety, agitation, and aggression.
4. Induce relaxation: helping patients to stay in a positive psychological state, which prevents emotional distress, makes it easier to cope with their situation, and improves the patients’ physical state (eg, through better sleep).
5. Provide a sense of security: supporting patients in feeling reassured and safe so that they can easily handle stress and emotional distress such as anxiety and fear that originate mainly from uncertainty and unfamiliarity.
6. Provide a sense of control: supporting patients by enhancing autonomy, empowerment, and control over anxiety.
7. Provide a sense of being connected: supporting patients to feel connected and socially engaged to prevent loneliness, depression, and anxiety.

Three of the 7 pathways outlined above, that is, *Restore the Circadian Rhythm, Activate the Body,* and *Activate the Mind,* are in line with strategies recommended in the ABCDEF bundle [16]. The other pathways are not directly linked, yet are associated with important predictors of well-being used in psychology: *Induce Relaxation* links to coping strategies [61] and *Provide a Sense of Security, Control,* and *Being Connected* are related to the universal psychological needs, which are security [62], dominance [63], and relatedness [62]. The 7 pathways, therefore, cover a broad range of delirium prevention strategies correlating physical, cognitive, and emotional aspects as shown in Figure 2.

## Discussion

### Overview

The results of this review provided an overview and characteristics of technology-based interventions that have been used to prevent and reduce delirium. We also summarized the related strategies into 7 pathways. These 7 pathways include key elements for developing technology-based interventions for delirium prevention. From our analysis of the included studies and technologies, we next discuss the limitations and opportunities for future research.

### Limitations in the Current Technology Use for Preventing Delirium

First, most technology-based interventions addressed only one or a few delirium risk factors. For instance, interventions that used a dynamic light system [41,60] aimed to improve the patients’ sleep-wake cycle while interventions, including video distraction [34,35,43], aimed to reduce the anxiety of the patients. Such approaches are not in line with the frequently recommended multicomponent approach, which effectively targets the multifactorial origin of delirium [15]. Second, most technology-based interventions were more momentary than continuous solutions. Although the length of stay in the ICU should be kept as short as possible, it can last from days to weeks. During this period, depending on the severity of their illness, patients are going through various clinical and emotional phases and their needs are changing respectively. During various activities (eg, clinical checkup, therapy session, cleaning, resting, family visit), patients can experience diverse feelings (eg, anxiety, fear, tiredness, worrying, relaxed, cheerful) and have different corresponding needs. In contrast to the heterogeneity of activities and patients’ needs throughout their ICU stay, the majority of the included studies focused on a short period of time. For instance, the video and video game interventions were usually planned for less than 5 minutes [34-36,43,50], and the VR interventions were used for less than 15 minutes [37,38,57]. In order to minimize delirium risk factors, it is important to understand the needs and concerns of patients throughout their whole ICU stay and to develop preventive solutions that are more continuous and seamless. Importantly, knowledge as how to develop and implement a continuous solution throughout the ICU stay of patients is missing as none of the reviewed studies evaluated it. Third, only a limited number of studies focused on the use of technology for improving the patients’ environment. Despite the negative influences of the stressors in the current ICU environment, such as overload of light and noise [64,65] on patients’ clinical progress, only a few studies [41,51,52,58,60] aim at optimizing the ICU environment. In contrast, most technologies used as interventions added extra sensory burden to the patients. Last, the effect of some technologies can be improved by a more thorough understanding of (vulnerable) patients and context-dependent needs. Understanding the patient’s needs is crucial for delivering high-quality care. However, sometimes, the implementation of technology can be challenging and might result in a suboptimal match with the patients and context. For instance, in the study of bright light therapy, patients were sedated and had their eyes closed and this resulted in insufficient amount of light exposure [41]. The use of wearable sleep-aid devices (earplugs and eye masks) by ICU patients can lead to potential discomfort, thereby limiting the effectiveness of the intervention [44].

### Further Development of Technology-Based Interventions for Preventing Delirium

Despite the limitations of current technology-based interventions, the potential of technology in delirium prevention is promising [20]. Reflecting on the identified limitations, the following recommendations are proposed for the future design and development of technology-based interventions for delirium prevention. First of all, technology should not only support physical and cognitive functions but also support psychological and emotional needs. In the analysis, the proposed 7 pathways described existing approaches for delirium prevention, which cover multifaceted needs of patients. Yet, comparing the number of related strategies and studies included in each pathway, it is notable that, in general, there are far more technology-based interventions aiming to support functions than to support needs. To take a more comprehensive approach, further development of technologies should aim at meeting patients’ needs by, for instance, providing a means to allow them more control over their situation, feel relaxed, safe, and connected. Some examples are an interaction device to enable ventilated patients to easily express their needs using gestures [66], a robotic pet that lets patients cuddle and helps them feel calm [67], or an intelligent alarm system contributing to a relaxing environment for ICU patients by controlling and harmonizing alarm sounds [68]. Another way to meet patients’ emotional and psychological needs can be found in the provision of (multi-) sensorial and cognitive stimuli. For instance, aromatherapy can be a way to support patients to feel relaxed through sensorial stimuli combining pleasant tactile pressure and aromatic fragrance [69]. For the stimuli, using nature elements can be an interesting candidate as exemplified by previous cases [43,57] and other applications: examples are a VR therapy showing various nature sceneries [70] and a geriatric care environment adapting nature elements [71] to generate relaxation [70,71], increase social engagement, and reduce restlessness [71]. Second, technology could create a healing environment for patients: a more context-aware, personalized, and adaptive ICU. Despite emerging interest in patient-centered care, this review showed that such interactive technology is rare in the current ICU environment. Patient-centered care recognizes a patient as a unique individual and stresses the importance of care tailored to patients’ specific preferences, needs, and values [72-74]. In order to better adapt patient-centered care, technology should be evolved in a way that (1) it allows patients to take a more active role in their care process, for instance, by enabling them to be explicit about their specific needs and (2) enables a care environment and service system to provide real-time interventions adapted to the patients’ profiles and their changing status/needs. Advances in technologies have made this feasible. Next to the modified ICU room [20] and the conversational agent [21] described in the introduction, the intelligent ICU concept enables autonomous monitoring of patients and the ICU environment over time by using pervasive sensing [75]. Previously we pointed out that there were too few technologies aiming at improving patients’ environment despite its significant influence on patients. Luetz et al [20] emphasized the potential of environment-related innovations, arguing that the ICU should be considered as a treatment tool. In order to design a healing environment, future technology-based interventions should reinforce the main ingredients of patient-centered care: context-awareness, personalization, and adaptability.

### Study Limitations

This study has some limitations. First, this review was conducted between 2015 and 2020 to focus on state-of-the-art technologies used for preventing and reducing delirium. Therefore, some technologies that might have been introduced in the studies published before 2015 and have not been studied since then were not included in this review. Second, the search strategy made use of the combination of keywords describing different types of technologies and not the keyword “technology” as this was not widely used in literature on delirium prevention and reduction. Although we tried to cover all the existing technology-related keywords, this search strategy might have left out some very rare types of technologies. Third, the 7 pathways were made based on the included studies only. Therefore, some approaches and strategies from the studies, which did not meet our criteria, were not included, such as approaches and strategies related to pharmacological interventions or delirium detection only.

### Conclusions

In this review, we provided an overview of technology-based interventions and proposed the 7 pathways to delirium prevention based on evidence-based studies. These insights can be considered as starting points for transforming ICUs into a healing environment, which might be well one of the most powerful nonpharmacological technology-based interventions for preventing delirium. Further research should generate a more in-depth and complete understanding of the key components of a healing environment for patients and on designing and developing technologies that can actualize it.

## Appendix

Multimedia Appendix 1 Search strategy.

Multimedia Appendix 2 Quality assessment.

Multimedia Appendix 3 Fourteen strategies used in the included studies.

## Abbreviations

ICU: intensive care unit
VR: virtual reality
