# Supplementary material for: Overview and Strategy Analysis of Technology-Based Nonpharmacological Interventions for In-Hospital Delirium Prevention and Reduction: Systematic Scoping Review
Source: J Med Internet Res. 2021 Aug 26;23(8):e26079. doi: 10.2196/26079 (PMC8430840; doi:10.2196/26079)
Supplement: Multimedia Appendix 1 [file jmir_v23i8e26079_app1.pdf]

Appendix 1: Search strategy

Database: Scopus <2015 to 2020 Jan 6>

1) (TITLE-ABS-KEY (deliri*) AND TITLE-ABS-KEY (technolog* OR intelligen* OR automat* OR computer OR computing OR robot* OR mobile OR app OR visual OR virtual OR vr OR video OR light* OR tracking OR ambien* OR game* OR sound* OR music* OR alarm OR (cognitive AND training) OR aroma* OR digital OR architect*)
2) 1 AND (LIMIT-TO (DOCTYPE , "ar") OR (LIMIT-TO (DOCTYPE , "cp")
3) 2 AND (LIMIT-TO (PUBYEAR , 2020) OR LIMIT-TO (PUBYEAR , 2019) OR LIMIT-TO (PUBYEAR , 2018) OR LIMIT-TO (PUBYEAR , 2017) OR LIMIT-TO (PUBYEAR , 2016) OR LIMIT-TO (PUBYEAR , 2015))
4) 3 AND (LIMIT-TO (LANGUAGE , "English")
