# Supplementary material for: Overview and Strategy Analysis of Technology-Based Nonpharmacological Interventions for In-Hospital Delirium Prevention and Reduction: Systematic Scoping Review
Source: J Med Internet Res. 2021 Aug 26;23(8):e26079. doi: 10.2196/26079 (PMC8430840; doi:10.2196/26079)
Supplement: Multimedia Appendix 2 [file jmir_v23i8e26079_app2.pdf]

Appendix 2: Quality assessment

Table S1 Methodological quality assessment scores of included studies

	Randomiz ation	Withdrawals Or dropouts	Control group	Appropriate size	Baseline similarity	**Total Score (0-5)**
**Arbabi 2018**	0	0	1	0	1	2
**Bott 2019**	0	0	1	1	0	2
**Byun 2018**	1	1	1	1	1	5
**Cheong 2016**	0	0	1	0	1	2
**Damshens 2018**	1	0	1	0	1	3
**Demoule 2017**	1	1	1	0	1	4
**Dwairej 2019**	1	1	1	1	1	5
**Eijlers 2019**	1	1	1	1	1	5
**Estrup 2018**	0	1	1	0	0	2
**Garry 2016**	0	1	0	0	0	1
**Giraud 2016**	1	1	1	0	1	4
**Johnson 2018**	1	1	1	1	1	5
**Kim 2015**	1	1	1	0	1	4
**Lee 2016**	0	0	1	1	1	3
**Lee 2017**	1	1	1	1	1	5
**Lin 2018**	1	1	1	1	1	5
**Mitchell 2017**	1	1	1	0	1	4
**Munro 2017**	1	1	1	0	0	3
**Potharajaroen 2018**	0	1	1	0	1	3
**Pustjens 2018**	0	0	1	0	1	2
**Rivosecchi 2016**	0	0	1	0	0	1
**Rodriguez 2019**	1	1	1	0	0	3
**Ryu 2018**	1	1	1	1	1	5
**Sharda 2018**	0	1	1	0	0	2
**Simons 2016**	1	1	1	0	0	3
**Smonig 2019**	0	0	1	1	1	3
**Suvajdzic 2019**	0	1	1	0	0	2
**Tovar 2016**	0	0	1	1	0	2
**Van de Pol 2017**	1	1 [a]	1	1	0	4
**Waszynski 2018**	1	1	1	0	1	4
**Zachary 2020**	1	0	1	0	1	3

[a] Due to the nature of intervention (measuring the sound of the environment), withdrawal was not relevant and the study described the number of patients before and after the intervention.

Table S2 The quality assessment system for included studies

Item	Score	Criteria
(1) Randomization	1	Randomization was described in an appropriate way.
	0	Randomization was not described or described but in an inappropriate way.
(2) Withdrawal or dropouts	1	Withdrawals or dropouts were described.
	0	Withdrawals or dropouts were not described.
(3) Control group	1	At least one control group (comparison) was included [a].
	0	No control group was included.
(4) Appropriate size	1	Sample size was justified (e.g., power calculation).
	0	Sample size was not justified.
(5) Baseline similarity	1	Delirium-related factors (demographics, prevalent delirium and other factors tested as baseline in study) were similar between groups.
	0	Delirium-related factors were different between groups or not described.

[a] Within-subject design was considered as having a control group.

*Due to the nature of most of technology-based interventions, which is often not possible to apply a double blinding setting, we did not include double blinding in our criteria.

References of the items

(1)-(4): Jadad AR, Moore RA, Carroll D, Jenkinson C, Reynolds DJM, Gavaghan DJ, et al. Assessing the Quality of Reports of Randomized Clinical Trials : Is Blinding Necessary ? Control Clin Trials. 1996;12(January 1995):1–12.

(5): Zhang H, Lu Y, Liu M, Zou Z, Wang L, Xu FY, et al. Strategies for prevention of postoperative delirium: a systematic review and meta-analysis of randomized trials. Crit Care. 2013;17(2).

O'Regan NA, Fitzgerald J, Timmons S, O'Connell H, Meagher D. Delirium : A key challenge for perioperative care. IJSU [Internet]. 2013;11(2):136–44. Available from: http://dx.doi.org/10.1016/j.ijsu.2012.12.015
