# Supplementary material for: Overview and Strategy Analysis of Technology-Based Nonpharmacological Interventions for In-Hospital Delirium Prevention and Reduction: Systematic Scoping Review
Source: J Med Internet Res. 2021 Aug 26;23(8):e26079. doi: 10.2196/26079 (PMC8430840; doi:10.2196/26079)
Supplement: Multimedia Appendix 3 [file jmir_v23i8e26079_app3.pdf]

Appendix 3

Table S1 Fourteen strategies used in the included technology-based interventions (✓– if a strategy is used)

		1. Cognitive stimulation & training	2. Companionship	3. Contextual cue (Reorientation)	4. Daytime awakening	5. Distraction	6. Early mobilization	7. Easier communication	8. Engagement	9. Familiarity	10. Good night sleep	11. Human interaction	12. Personalization	13. Psychological preparation	14. Soothing Elements
Music	Damshens [b] [40]	✓													
	Lee et al[b] [39]														✓
	Johnson et al[c] [48]														✓
	Sharda et al[a] [53]									✓			✓		✓
	Cheong et al[a] [55]								✓	✓					✓
Voice	Byun et al[a] [33]			✓						✓					
	Munro et al[a] [49]			✓						✓					
Light	Estrup et al[b] [58]				✓										
	Pustjens et al[b] [60]				✓										
	Simons et al[a] [41]				✓						✓				
	Potharajaroen et al[b][42]				✓										
	Smonig et al[b] [54]				✓										
Video	Lee et al[b] [50]													✓	
	Kim et al[a] [34]					✓									
	Rodriguez et al[a] [35]					✓									
	Waszynski et al[a] [43]									✓					✓
	Dwairej et al[a] [36]					✓									
Virtual reality	Eijlers et al[a] [37]													✓	
	Ryu et al[a] [38]													✓	
	Suvajdzic et al[a] [57]					✓									✓
Sleep	Demoule et al[a] [44]										✓				
	Van de Pol et al[a] [19]										✓				
Communication	Garry et al[a] [56]							✓							
	Bott et al [b] [21]		✓					✓				✓			
Others	Lin et al[b] [46]													✓	
	Giraud et al[a] [45]	✓					✓	✓							
Multiple components	Arbabi et al[b] [51]			✓							✓				
	Tovar et al[a] [52]	✓								✓	✓				✓
	Rivosecchi et al[a] [24]	✓		✓	✓										✓
	Mitchell et al[a] [47]	✓		✓								✓			
	Zachary et al[a] [59]	✓		✓							✓				✓

[a]Strategy being explicitly mentioned by authors.
[b]Strategy being used but not explicitly mentioned by authors.
[c]Interpretation of strategy was made: activating physiological reaction (author's words) by using soothing elements.
